# Early Changes in Spleen Stiffness After Endoscopic Treatment Are Associated with Esophageal Variceal Recurrence: A Pilot Study

**DOI:** 10.3390/jcm15207868

**Published:** 2026-10-12

**Authors:** Akira Uchiyama, Shunhei Yamashina, Satoshi Sakuma, Masahiro Tada, Kei Ishizuka, Maki Morinaga, Hiroo Fukada, Reiko Yaginuma, Kyoko Fukuhara, Kazuyoshi Kon, Kenichi Ikejima

**Affiliations:** Department of Gastroenterology, School of Medicine, Juntendo University, 2-1-1 Hongo, Bunkyo-Ku, Tokyo 113-8421, Japan; syamashi@juntendo.ac.jp (S.Y.); s.sakuma.dn@juntendo.ac.jp (S.S.); h-fukada@juntendo.ac.jp (H.F.); yagirei@juntendo.ac.jp (R.Y.); kyokof@juntendo.ac.jp (K.F.); kazukon@juntendo.ac.jp (K.K.); ikejima@juntendo.ac.jp (K.I.)

**Keywords:** spleen stiffness, elastography, esophageal varices, variceal recurrence

## Abstract

**Background:** Spleen stiffness (SS) is a noninvasive marker of portal hypertension; however, the clinical significance of early changes in SS after endoscopic treatment for esophageal varices remains unclear. We evaluated whether SS and its early post-treatment changes are associated with variceal recurrence. **Methods:** This retrospective pilot study included 20 patients who underwent endoscopic treatment for esophageal varices. Liver stiffness (LS) and spleen stiffness (SS) were measured before treatment, approximately 1 week after the final treatment session (1W), and at 1 month and 1 year after treatment. The clinical outcome of interest was variceal recurrence within 1 year. Early changes in stiffness were calculated from 1W to 1M (Δ1M − 1W). **Results:** Eleven patients (55.0%) developed variceal recurrence. LS did not differ significantly between the non-recurrence and recurrence groups at any time point during follow-up. SS was comparable between the groups before treatment and at 1W but was significantly higher in the recurrence group at 1M (median [IQR], 2.75 [2.70–2.86] vs. 3.40 [3.06–3.81] m/s in the non-recurrence and recurrence groups, respectively; *p* = 0.003). ΔSS-VTQ (1M − 1W) was significantly greater in the recurrence group than in the non-recurrence group (−0.13 [−0.62–−0.04] vs. 0.25 [−0.05–0.40] m/s, respectively; *p* = 0.013), whereas no significant between-group differences were observed in changes in LS. **Conclusions:** SS at 1 month after endoscopic treatment and its early post-treatment change were associated with variceal recurrence. Serial assessment of SS may provide useful information for early post-treatment risk assessment, although validation in larger prospective studies is needed.

## 1. Introduction

Accurate assessment of portal hypertension plays a pivotal role in determining the prognosis and guiding therapeutic strategies in patients with compensated advanced chronic liver disease [1]. The hepatic venous pressure gradient (HVPG) is the reference standard for assessing portal hypertension and provides important prognostic information regarding hepatic decompensation and other portal hypertension-related outcomes [2]. Patients with an HVPG > 10 mmHg are at increased risk of hepatic decompensation; therefore, accurate diagnosis of clinically significant portal hypertension (CSPH) is important [3]. However, HVPG measurement is invasive and available only in specialized centers, limiting its widespread clinical applicability.

Esophagogastroduodenoscopy (EGD) remains the gold standard for the detection and assessment of EV [4]. The Japanese guidelines for liver cirrhosis recommend endoscopic treatment for EV at high risk of bleeding, with red color signs representing an important endoscopic indicator of rupture risk [5,6]. Endoscopic treatment includes endoscopic variceal ligation (EVL) and endoscopic injection sclerotherapy (EIS) [7,8]. The Baveno VI consensus introduced liver stiffness (LS) measured by transient elastography (TE), together with platelet count, as a noninvasive approach to identify patients at low risk of high-risk varices who may avoid screening endoscopy [9]. However, LS can be influenced by the etiology of liver disease and other factors unrelated to portal hypertension, limiting its ability to directly reflect portal hemodynamics [10]. More recently, spleen stiffness (SS) has emerged as a promising noninvasive marker that may more closely reflect portal hypertension. Accordingly, SS measurement has been incorporated into the EASL guidelines for the noninvasive assessment and risk stratification of CSPH and EV. The recently published Baveno VIII consensus further emphasized the role of noninvasive tests in the assessment of CSPH and recognized spleen stiffness measurement as a tool that may further improve the classification of CSPH [1,11,12].

Spleen stiffness can be assessed using several elastography techniques, including TE and Virtual Touch Quantification (VTQ). Previous studies have demonstrated good diagnostic performance of SS for identifying varices needing treatment [13]. However, most studies have focused on the presence or severity of varices, and little is known about changes in SS after endoscopic treatment. In particular, whether early post-treatment changes in SS are associated with subsequent variceal recurrence remains unclear. Therefore, this pilot study aimed to evaluate longitudinal changes in LS and SS after endoscopic treatment, with particular focus on the association between early changes in SS and variceal recurrence.

## 2. Methods

### 2.1. Study Design and Setting

**Figure 1:**
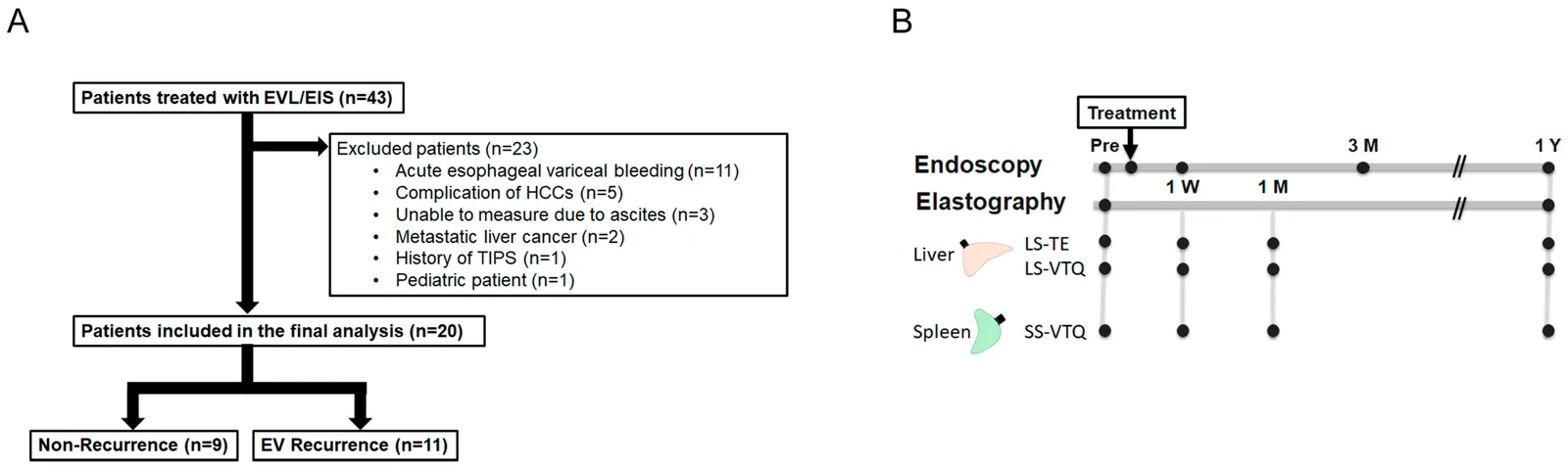
**Patient selection and study protocol.** (**A**) Flow diagram of patient enrollment and selection. Of 43 patients with EV who underwent EVL or EIS during the study period, 23 were excluded because of acute esophageal variceal bleeding (*n* = 11), complications of HCC (*n* = 5), inability to measure stiffness due to ascites (*n* = 3), metastatic liver cancer (*n* = 2), history of TIPS (*n* = 1), or pediatric age (*n* = 1). The remaining 20 patients were included in the final analysis and were classified into the non-recurrence (*n* = 9) and recurrence (*n* = 11) groups according to EV recurrence within 1 year. (**B**) Schedule of endoscopic examinations and serial liver and spleen stiffness measurements. Endoscopy was performed before treatment (Pre), approximately 1 week after the final treatment session (1W) to confirm variceal eradication, and at 3 months (3M) and 1 year (1Y) after treatment. LS-TE, LS-VTQ, and SS-VTQ were measured before treatment and at 1W, 1 month (1M), and 1Y after treatment. Abbreviations: EV, esophageal varices; EVL, endoscopic variceal ligation; EIS, endoscopic injection sclerotherapy; HCC, hepatocellular carcinoma; TIPS, transjugular intrahepatic portosystemic shunt; LS-TE, liver stiffness measured by transient elastography; LS-VTQ, liver stiffness measured by Virtual Touch Quantification; SS-VTQ, spleen stiffness measured by Virtual Touch Quantification.

The study protocol was approved by the Institutional Ethics Committee of Juntendo University Hospital (approval code: E25-0380). This was a single-center pilot cohort study based on a retrospective analysis of prospectively collected elastography data, conducted at the Department of Gastroenterology, Juntendo University Hospital (Tokyo, Japan). Liver and spleen stiffness measurements were prospectively scheduled at predefined time points before endoscopic treatment, approximately 1 week after the final treatment session, and 4–5 weeks after the final treatment session (1-month [1M]). Patients were identified as candidates for endoscopic treatment based on diagnostic EGD, and baseline stiffness measurements were obtained before the scheduled EVL/EIS procedure. A total of 43 patients with EV who underwent EVL or EIS between January 2018 and January 2019 and had liver and spleen stiffness measurements available at these predefined time points were screened for eligibility. As shown in Figure 1A, 23 patients were excluded according to the predefined exclusion criteria: acute esophageal variceal bleeding (*n* = 11), complications of hepatocellular carcinoma (*n* = 5), inability to undergo stiffness measurement due to ascites (*n* = 3), metastatic liver cancer (*n* = 2), history of transjugular intrahepatic portosystemic shunt (TIPS) (*n* = 1), and pediatric age (*n* = 1). The remaining 20 patients were included in the final analysis. During follow-up, EV recurrence was observed in 11 patients, whereas 9 patients had no recurrence.

### 2.2. Clinical and Imaging Assessments

Baseline clinical and laboratory data, including age, sex, etiology of liver disease, platelet count (Plt), prothrombin time–international normalized ratio (PT-INR), alanine aminotransferase (ALT), aspartate aminotransferase (AST), albumin (Alb), and ammonia (NH3), were collected for each patient. The Child–Pugh score was calculated to assess liver function. The splenic index was assessed by computed tomography (CT) before treatment and at 1 year after treatment. Dynamic CT was performed with 5 mm slice thickness, and portal-phase images obtained before treatment were used to evaluate the presence of paraesophageal veins and portosystemic shunts, including splenorenal, paraumbilical, and gonadal shunts.

### 2.3. Endoscopic Treatment and Assessment of Variceal Recurrence

Endoscopic treatment was performed by specialists certified by the Japanese Society of Gastroenterological Endoscopy and the Japan Society of Hepatology. Esophageal varices were classified as Form 1 (small and straight), Form 2 (enlarged and tortuous, occupying less than one-third of the esophageal lumen), or Form 3 (large and coil-shaped, occupying more than one-third of the esophageal lumen). Treatment with endoscopic injection sclerotherapy (EIS) or endoscopic variceal ligation (EVL) was selected according to the endoscopic characteristics of the varices [14,15]. EIS was performed using 5% ethanolamine oleate (5%EO; OLDAMIN^®^, ASKA Pharmaceutical Co., Ltd., Tokyo, Japan) as the sclerosant, with a maximum total dose of 0.4 mL/kg per procedure [8].

Red color signs (RC signs) included red wale markings, cherry-red spots, and hematocystic spots, according to the Japanese classification of esophageal varices [15]. EVL was generally completed in a single treatment session, whereas EIS was performed in multiple treatment sessions at approximately 1-week intervals until variceal eradication was achieved. Approximately 1 week after completion of endoscopic treatment, EGD was performed to assess the treatment response, and eradication of esophageal varices (F0, RC0) was confirmed before discharge. Thereafter, follow-up EGD was performed at 3 months and 1 year to assess variceal recurrence (Figure 1B). Recurrence was defined as the development of Form 2 or greater varices or the presence of RC signs on follow-up endoscopy [15]. The schedule of endoscopic examinations and stiffness measurements is shown in Figure 1B.

### 2.4. Liver and Spleen Stiffness Measurements

Patients underwent stiffness measurements after fasting. Transient elastography (TE; FibroScan^®^, Echosens, Paris, France) and Virtual Touch Quantification (VTQ; ACUSON 2000^®^, Siemens, Issaquah, WA, USA) were used to assess liver and spleen stiffness [9,16]. Liver stiffness measured by TE (LS-TE), liver stiffness measured by VTQ (LS-VTQ), and spleen stiffness measured by VTQ (SS-VTQ) were assessed by board-certified gastroenterologists experienced in abdominal ultrasonography and elastography.

VTQ uses acoustic radiation force impulse (ARFI) technology to quantify tissue stiffness by measuring shear wave velocity (SWV) in the liver and spleen. For liver stiffness measurements, the FibroScan^®^ probe was positioned intercostally at sites corresponding to the VTQ measurement locations. For SS-VTQ measurements, the ACUSON ultrasound probe was positioned at the inferior pole of the spleen. To minimize measurement variability during serial assessments, previous examinations were referenced whenever possible. For LS-VTQ and SS-VTQ, the region of interest (ROI) was placed at approximately the same anatomical location and depth as in the previous examination, and LS-TE was performed through the same intercostal space whenever feasible. LS-TE was expressed as the median of at least 10 valid measurements, whereas SS-VTQ was expressed as the median of at least four valid measurements. Measurements were performed by experienced operators who were blinded to subsequent variceal recurrence. LS-TE, LS-VTQ, and SS-VTQ were assessed before endoscopic treatment (Pre) and at 1 week (1W), 1 month (1M), and 1 year (1Y) after treatment, as shown in Figure 1B. The 1W measurement was performed approximately 1 week after the final treatment session, at the time of endoscopic confirmation of variceal eradication, and was considered the early post-treatment reference point for evaluating subsequent changes in liver and spleen stiffness from 1W to 1M. Analyses at each time point were performed using the available measurements.

### 2.5. Statistical Analysis

The normality of continuous variables was assessed using the Shapiro–Wilk test. Given the small sample size of this pilot study, continuous variables are presented as median (interquartile range [IQR]) and were compared between the recurrence and non-recurrence groups using the Mann–Whitney U test. Categorical variables are expressed as counts (percentages) and were compared using Fisher’s exact test or the Fisher–Freeman–Halton exact test. A two-sided *p* value < 0.05 was considered statistically significant. Given the pilot and exploratory nature of this study, a single primary comparison was not prespecified, and no formal adjustment for multiple comparisons was performed. Accordingly, the reported *p* values should be interpreted as exploratory rather than confirmatory. To provide estimates of the magnitude and precision of the observed differences, Hodges–Lehmann estimates of the between-group location shift and their 95% confidence intervals (CIs) were calculated for SS-VTQ at 1 month and the change in SS-VTQ from 1 week to 1 month (ΔSS-VTQ), with the estimated differences expressed as recurrence minus non-recurrence. All statistical analyses were performed using SigmaPlot version 15.5 (Systat Software Inc., San Jose, CA, USA), with the Hodges–Lehmann estimates and their 95% CIs additionally calculated using R version 4.2.0 (R Foundation for Statistical Computing, Vienna, Austria).

## 3. Results

### 3.1. Baseline Patient Characteristics

Twenty patients were included in the final analysis (Figure 1A), of whom eleven (55.0%) developed variceal recurrence within 1 year. Of these, four met the recurrence criterion at the 3-month follow-up EGD, whereas the remaining seven met the criterion at the 1-year follow-up EGD. Among the four patients with recurrence detected at 3 months, two underwent additional EIS, one underwent partial splenic embolization (PSE), and one underwent balloon-occluded retrograde transvenous obliteration (BRTO). All 20 patients completed the scheduled 1-year endoscopic follow-up, with no loss to follow-up, deaths, liver transplantation, or other events that precluded assessment of EV recurrence. No variceal bleeding occurred during the 1-year follow-up period. Portal hypertension-related complications developed in three patients: ascites in two and hepatic encephalopathy in one. EVL was completed in a single treatment session in all patients, with a median of 7.5 ligation bands (IQR, 6.25–8) used per patient. EIS required a median of 3 treatment sessions (IQR, 3–3), with a median total volume of 5% EO of 11 mL (IQR, 3–14) per patient. The interval from the initial treatment session to endoscopic confirmation of variceal eradication was 7 days for EVL and 21 days for EIS. Baseline characteristics of the recurrence and non-recurrence groups are summarized in Table 1. No statistically significant between-group differences were detected in age, sex, etiology of liver disease, variceal grade, treatment modality (EVL vs. EIS), splenic index, laboratory parameters, or Child–Pugh score. The underlying etiologies were HCV (*n* = 4), MASLD (*n* = 5), ALD (*n* = 7), autoimmune liver disease (*n* = 3), and EHPVO (*n* = 1). All seven patients with ALD maintained complete abstinence from alcohol after endoscopic treatment. All patients except the patient with EHPVO had established liver cirrhosis. No patients had overlapping etiologies of liver disease. No patients received nonselective beta-blockers at baseline or during the 1-year follow-up period because of hypotension.

### 3.2. Serial Changes in Liver and Spleen Stiffness After Endoscopic Treatment

No statistically significant between-group differences were observed in LS-TE or LS-VTQ at any time point during follow-up. Similarly, SS-VTQ was comparable between the non-recurrence and recurrence groups before treatment (2.76 [2.74–3.17] vs. 3.40 [3.35–3.59] m/s, respectively; *p* = 0.128) and at 1W (3.08 [2.85–3.36] vs. 3.30 [2.90–3.47] m/s, respectively; *p* = 0.583). At 1M, SS-VTQ was higher in the recurrence group than in the non-recurrence group (3.40 [3.06–3.81] vs. 2.75 [2.70–2.86] m/s; Hodges–Lehmann estimated difference, 0.65 m/s [95% CI, 0.20–1.04]; *p* = 0.003). At 1Y, SS-VTQ remained numerically higher in the recurrence group (3.06 [2.93–3.46] vs. 2.83 [2.62–3.07] m/s), although the between-group difference was not statistically significant (*p* = 0.127).

**Figure 2:**
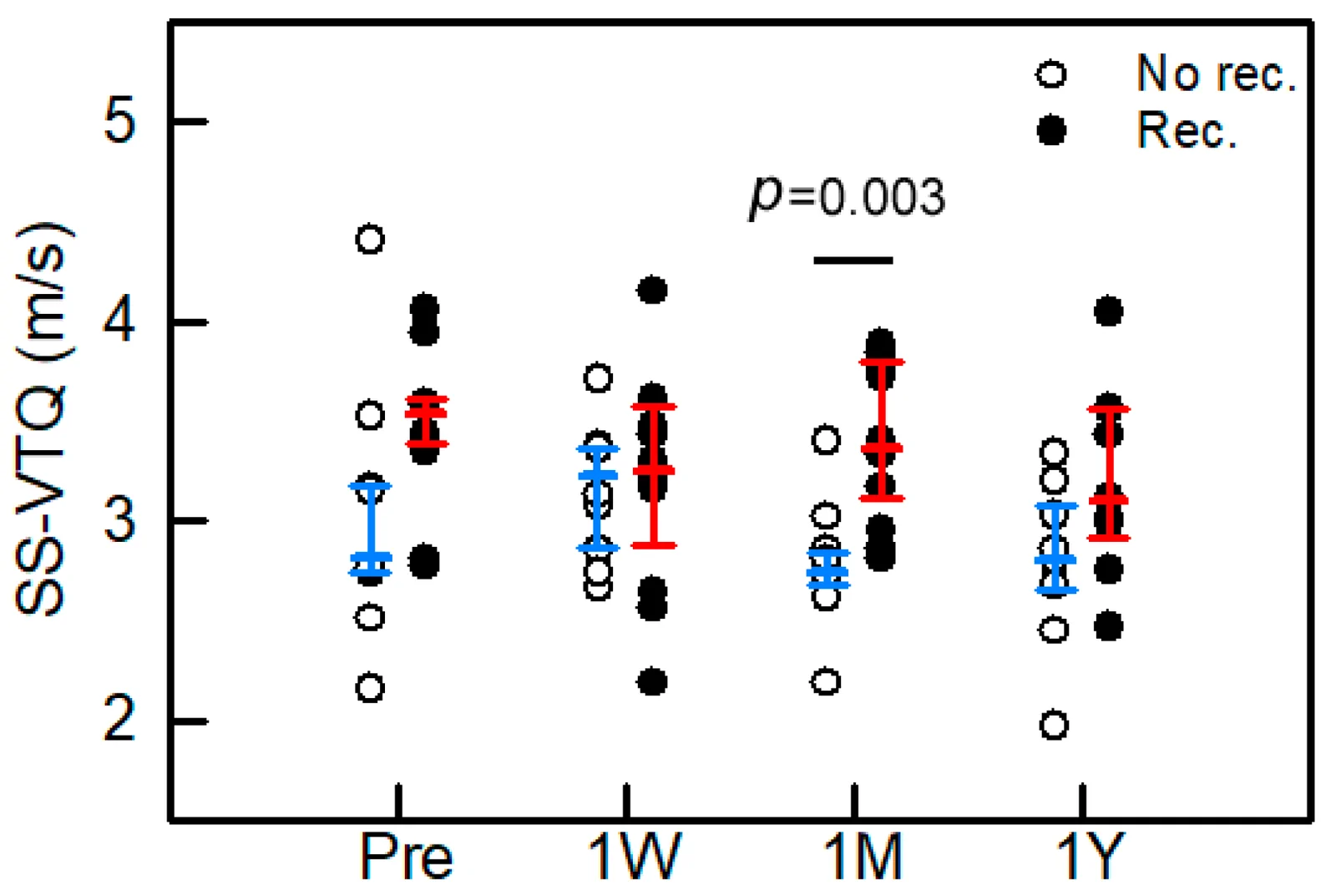
**Longitudinal changes in spleen stiffness measured by VTQ after treatment.** Individual SS-VTQ values are shown for the non-recurrence (open circles) and recurrence (closed circles) groups before treatment (Pre) and at 1 week (1W), 1 month (1M), and 1 year (1Y) after treatment. The blue and red lines represent the non-recurrence and recurrence groups, respectively. Horizontal bars and error bars indicate the median and interquartile range, respectively. Between-group comparisons at each time point were performed using the Mann–Whitney U test. A significant difference between the groups was observed at 1M (*p* = 0.003).

The longitudinal profiles of individual SS-VTQ values in the two groups are shown in Figure 2. Consistent with the findings presented in Table 2, no statistically significant between-group differences in SS-VTQ were observed before treatment or at 1W, whereas higher SS-VTQ values were observed in the recurrence group at 1M. At 1Y, SS-VTQ remained numerically higher in the recurrence group, although the difference was not statistically significant. Similarly, no statistically significant between-group differences were observed in LS-TE or LS-VTQ at 1M (Supplemental Figure S1A,B).

### 3.3. Early Post-Treatment Changes in Spleen Stiffness and EV Recurrence

**Figure 3:**
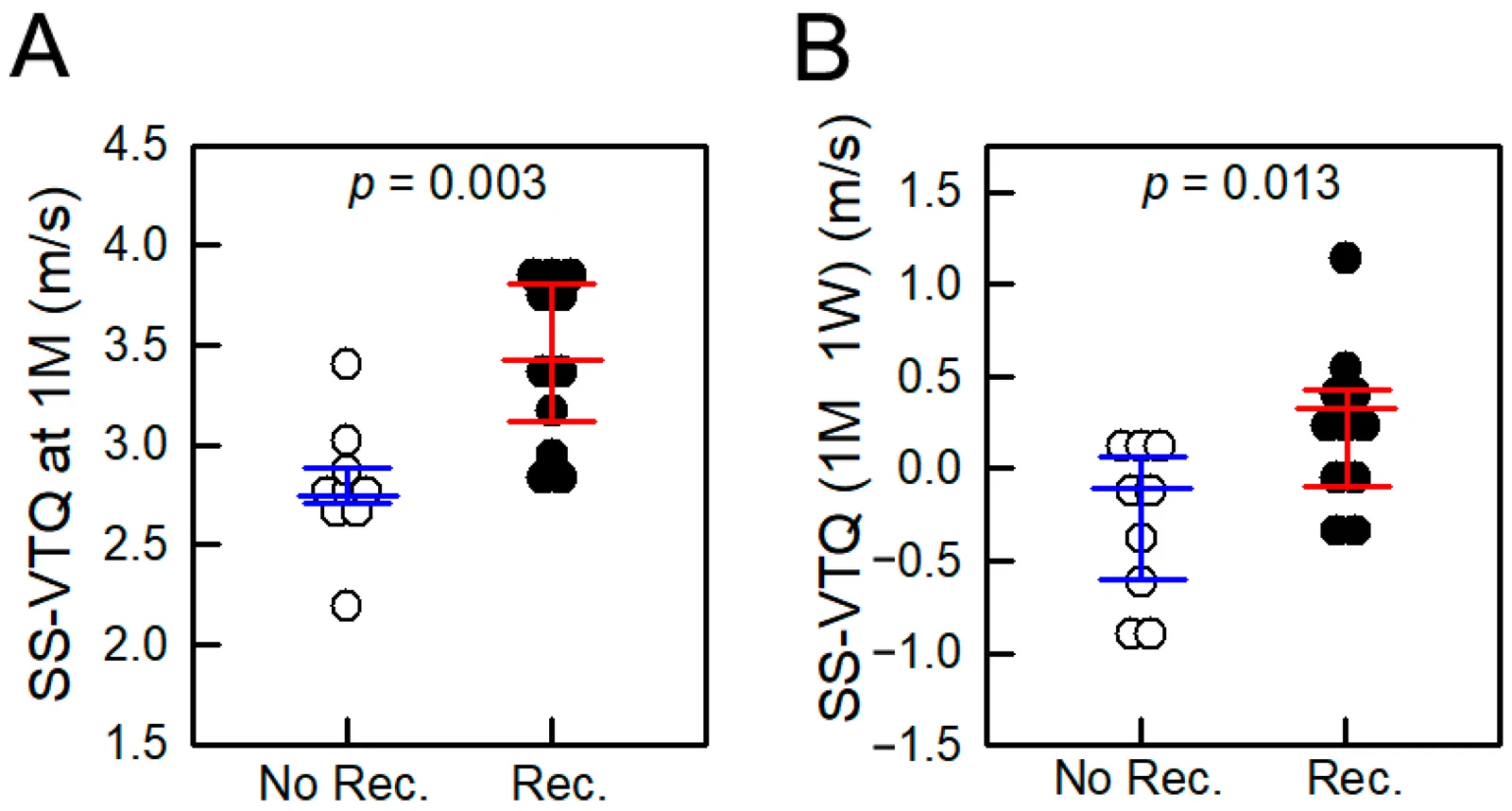
Spleen stiffness at 1 month and its early post-treatment change according to esophageal variceal recurrence. (**A**) Distribution of SS-VTQ values at 1 month (1M) in the non-recurrence and recurrence groups. (**B**) Distribution of changes in SS-VTQ from 1 week to 1 month (ΔSS-VTQ [1M − 1W]) in the non-recurrence and recurrence groups. Open circles represent the non-recurrence group, and closed circles represent the recurrence group. The blue and red lines represent the non-recurrence and recurrence groups, respectively. Horizontal bars and error bars indicate the median and interquartile range, respectively. Between-group comparisons were performed using the Mann–Whitney U test. SS-VTQ at 1M was higher in the recurrence group than in the non-recurrence group (*p* = 0.003), and ΔSS-VTQ (1M − 1W) was greater in the recurrence group (*p* = 0.013).

We next focused on SS-VTQ at 1M and the early post-treatment change in stiffness from 1W to 1M. Because the 1W measurement represented an early post-treatment time point after variceal eradication had been achieved, we used this time point as the reference to evaluate subsequent changes up to 1M. The distribution of individual SS-VTQ values at 1M is shown in Figure 3A.

Changes in stiffness parameters from 1W to 1M (1M − 1W) were then evaluated. As shown in Table 3, ΔSS-VTQ (1M − 1W) was greater in the recurrence group than in the non-recurrence group (median [IQR], 0.25 [−0.05–0.40] vs. −0.13 [−0.62–−0.04] m/s; Hodges–Lehmann estimated difference, 0.47 m/s [95% CI, 0.08–0.97]; *p* = 0.013). In contrast, no statistically significant between-group differences were observed in ΔLS-TE (1M − 1W) or ΔLS-VTQ (1M − 1W). The distribution of ΔSS-VTQ (1M − 1W) is shown in Figure 3B.

### 3.4. Splenic Index and Portosystemic Shunts

Changes in the splenic index from baseline to 1 year after treatment did not significantly differ between the recurrence and non-recurrence groups (Supplemental Figure S2). In contrast, portosystemic shunts were significantly more frequent in the non-recurrence group than in the recurrence group (66.7% [6/9] vs. 9.1% [1/11], respectively; *p* = 0.017; Supplemental Table S1). Paraesophageal veins were also more frequently observed in the non-recurrence group, although the difference did not reach statistical significance (66.7% [6/9] vs. 27.3% [3/11], respectively; *p* = 0.175).

## 4. Discussion

The present pilot study demonstrated that spleen stiffness measured by VTQ at 1 month after endoscopic treatment and its early post-treatment change from 1 week to 1 month (ΔSS-VTQ) were associated with variceal recurrence. Notably, ΔSS-VTQ differed significantly between patients with and without recurrence, with spleen stiffness tending to decrease in the non-recurrence group but increase in the recurrence group during this period (Figure 2 and Figure 3B). These findings suggest that, in addition to spleen stiffness measured at a single time point, early dynamic changes in spleen stiffness after treatment may provide additional information related to subsequent variceal recurrence. However, although efforts were made to minimize measurement variability during serial assessments, some degree of variability cannot be completely excluded and should be considered when interpreting the short-term changes in SS-VTQ from 1W to 1M.

The early post-treatment period may be particularly important because changes in spleen stiffness during this period may reflect alterations in portal hemodynamics following endoscopic treatment. At 1 week after treatment, the treated varices had been eradicated, providing an early post-treatment reference point from which subsequent changes in spleen stiffness could be evaluated. Notably, SS-VTQ tended to decrease in the non-recurrence group, whereas it tended to increase in the recurrence group from 1 week to 1 month. These differential changes may reflect differences in early post-treatment portal hemodynamics associated with subsequent variceal recurrence. No corresponding between-group difference in liver stiffness was detected in this small cohort. Whether spleen stiffness is more responsive than liver stiffness to early post-treatment changes requires further study. Previous studies have demonstrated a close relationship between spleen stiffness and portal hypertension [17,18,19,20].

Although the splenic index has been reported to be useful for detecting the presence of esophageal varices [21,22], no significant difference in the change in splenic index from baseline to 1 year after treatment was observed between the recurrence and non-recurrence groups in the present study (Supplementary Figure S2). This finding suggests that the observed differences in spleen stiffness may not simply reflect changes in splenic size. However, because the splenic index was assessed only at baseline and 1 year after treatment, its temporal changes cannot be directly compared with the early changes in spleen stiffness observed from 1 week to 1 month.

The potential influence of portosystemic collateral circulation on spleen stiffness should also be considered. Portosystemic shunts were significantly more frequent in the non-recurrence group than in the recurrence group (Supplementary Table S1). Such shunts may modify portal hemodynamics by diverting portal blood flow into the systemic circulation [20,23], potentially reducing the hemodynamic burden on the spleen. This finding may therefore provide a possible hemodynamic context for the lower spleen stiffness observed in patients without recurrence. However, given the marked imbalance in the prevalence of portosystemic shunts between the two groups, their potential confounding effect on the observed association between spleen stiffness and EV recurrence cannot be excluded. In an exploratory comparison, ΔSS-VTQ did not differ significantly according to the presence or absence of portosystemic shunts. Moreover, our assessment was limited to the presence or absence of major portosystemic shunts and did not include quantitative evaluation of collateral flow or detailed collateral characteristics. Therefore, the relationship between collateral circulation, post-treatment changes in spleen stiffness, and variceal recurrence remains uncertain. Spleen stiffness has emerged as a useful noninvasive marker of portal hypertension and has been incorporated into recent consensus recommendations, including the Baveno consensus [1,12,24]. From a clinical perspective, serial assessment of spleen stiffness may provide additional information regarding the risk of variceal recurrence after endoscopic treatment. Ultrasound elastography allows noninvasive and repeated assessment of spleen stiffness and is therefore well suited for longitudinal follow-up. In particular, our findings suggest that changes in spleen stiffness during the early post-treatment period may help identify patients who warrant closer endoscopic surveillance.

This study has several limitations. First, the small, single-center sample limited statistical power, particularly for liver stiffness analyses, and precluded reliable multivariable adjustment. External validation was not performed. Second, the screened population was limited to patients with serial liver and spleen stiffness measurements available at the predefined time points, rather than all consecutive patients undergoing EVL or EIS during the study period; therefore, selection bias cannot be excluded. Third, direct portal pressure measurements were unavailable; therefore, the hemodynamic interpretation of changes in spleen stiffness remains inferential. Fourth, portosystemic shunts were more frequent in the non-recurrence group and may have confounded the association between spleen stiffness and EV recurrence. Fifth, the cohort included patients with different liver disease etiologies and one patient with extrahepatic portal vein obstruction. Sixth, additional treatment after recurrence at 3 months may have affected the 1-year stiffness measurements. Finally, no primary comparison was prespecified or adjustment made for multiple testing, increasing the possibility of chance findings; clinically useful thresholds also remain to be established. These findings are therefore exploratory and require confirmation in larger prospective studies.

## 5. Conclusions

Spleen stiffness at 1 month after endoscopic treatment and its early post-treatment change were associated with variceal recurrence. These hypothesis-generating findings suggest that serial assessment of spleen stiffness may provide useful information for post-treatment risk assessment. Larger prospective studies are needed to determine whether serial spleen stiffness measurements provide additional value beyond established clinical and endoscopic factors.

## Figures and Tables

**Table 1:** Baseline characteristics of patients in the recurrence and non-recurrence groups.

	All (*n* = 20)	Non-Recurrence Group (*n* = 9)	Recurrence Group (*n* = 11)	*p* Value
Age, years, median (IQR)	64.5 (51.8–70.3)	67.0 (62.0–71.0)	61.0 (51.0–69.5)	0.689
Male sex, *n*/N (%)	11/20 (55.0)	4/9 (44.4)	7/11 (63.6)	0.653
Etiology of Liver disease *n*/N (%) HCV MASLD ALD Autoimmune liver disease EHPVO	4/20 (20.0) 5/20 (25.0) 7/20 (35.0) 3/20 (15.0) 1/20 (5.0)	2/9 (22.2) 3/9 (33.3) 3/9 (33.3) 1/9 (11.1) 0/9 (0)	2/11 (18.2) 2/11 (18.2) 4/11 (36.4) 2/11 (18.2) 1/11 (9.1)	1.000
Variceal grade F2, *n*/N (%)	18/20 (90.0)	9/9 (100)	9/11 (81.8)	0.479
EVL, *n*/N (%)	15/20 (75.0)	7/9 (77.8)	8/11 (72.7)	1.000
Use of NSBB, *n*/N (%)	0/20 (0)	0/9 (0)	0/11 (0)	
Splenic index, median (IQR)	57.9 (47.5–63.3)	58.0 (42.2–60.5)	57.7 (51.1–73.9)	0.382
PLT (×10^4^/µL)	9.2 (6.3–10.8)	9.7 (9.1–12.6)	6.9 (5.9–10.1)	0.210
PT-INR	1.12 (1.08–1.18)	1.15 (1.10–1.18)	1.12 (1.08–1.17)	0.995
AST (IU/L)	35 (31–50)	37 (34–51)	33 (30–45)	0.470
ALT (IU/L)	30 (22–35)	31 (30–35)	26 (20–34)	0.458
Alb (g/dL)	3.8 (3.6–4.0)	3.8 (3.5–3.9)	3.8 (3.7–4.2)	0.575
NH_3_ (μg/dL)	45 (36.5–52.5)	44 (35–57)	46 (39–48)	0.967
Child–Pugh score	5 (5–5)	5 (5–5)	5 (5–5)	0.827

Continuous variables are presented as median (interquartile range [IQR]) and were compared using the Mann–Whitney U test. Categorical variables are presented as *n*/N (%) and were compared using Fisher’s exact test or the Fisher–Freeman–Halton exact test, as appropriate. A *p* value < 0.05 was considered statistically significant. Autoimmune liver disease included primary biliary cholangitis (*n* = 1), autoimmune hepatitis (*n* = 1), and primary sclerosing cholangitis (*n* = 1). AIH, autoimmune hepatitis; PBC, primary biliary cholangitis; PSC, primary sclerosing cholangitis. Abbreviations: HCV, hepatitis C virus; MASLD, metabolic dysfunction-associated steatotic liver disease; ALD, alcohol-associated liver disease; EHPVO, extrahepatic portal vein obstruction; EVL, endoscopic variceal ligation; EIS, endoscopic injection sclerotherapy; NSBB, nonselective beta-blockers; PLT, platelet count; PT-INR, prothrombin time–international normalized ratio; AST, aspartate aminotransferase; ALT, alanine aminotransferase; Alb, albumin; NH3, ammonia.

**Table 2:** Liver and spleen stiffness measurements.

		All (*n* = 20)	Non-Recurrence Group (*n* = 9)	Recurrence Group (*n* = 11)	*p* Value
Pre	LS-TE (kPa)	17.5 (9.9–22.5)	17.6 (10.5–21.8)	14.8 (10.1–22.7)	0.786
	LS-VTQ (m/s)	2.51 (2.10–3.16)	2.40 (2.10–2.87)	2.55 (2.18–3.21)	0.941
	SS-VTQ (m/s)	3.35 (2.77–3.54)	2.76 (2.74–3.17)	3.40 (3.35–3.59)	0.128
1W	LS-TE (kPa)	17.3 (11.1–21.9)	12.9 (10.9–24.2)	18.2 (11.9–19.6)	0.870
	LS-VTQ (m/s)	2.75 (2.36–2.97)	2.84 (2.67–2.94)	2.58 (2.26–2.98)	0.409
	SS-VTQ (m/s)	3.19 (2.82–3.44)	3.08 (2.85–3.36)	3.30 (2.90–3.47)	0.583
1M	LS-TE (kPa)	18.6 (12.2–27.0)	18.6 (12.4–27.0)	18.4 (12.9–25.2)	0.902
	LS-VTQ (m/s)	2.43 (1.98–2.90)	2.57 (2.11–2.97)	2.10 (1.97–2.86)	0.369
	SS-VTQ (m/s)	2.99 (2.80–3.48)	2.75 (2.70–2.86)	3.40 (3.06–3.81)	0.003
1Y	LS-TE (kPa)	13.9 (12.0–18.2)	13.9 (12.4–18.0)	14.6 (12.0–18.2)	0.923
	LS-VTQ (m/s)	2.28 (2.20–2.61)	2.35 (2.15–2.61)	2.25 (2.23–2.61)	0.897
	SS-VTQ (m/s)	3.00 (2.73–3.24)	2.83 (2.62–3.07)	3.06 (2.93–3.46)	0.127

LS-TE measurements were available for 18 patients; LS-VTQ and SS-VTQ measurements were available for all 20 patients. Continuous variables are expressed as median (interquartile range) and were compared using the Mann–Whitney U test. A *p* value < 0.05 was considered statistically significant. Abbreviations: LS-TE, liver stiffness-transient elastography; LS-VTQ, liver stiffness-Virtual Touch Quantification; SS-VTQ, spleen stiffness-Virtual Touch Quantification.

**Table 3:** Early post-treatment changes in liver and spleen stiffness according to esophageal variceal recurrence.

	All	Non-Recurrence Group	Recurrence Group	*p* Value
ΔLS-TE (1M − 1W), *n* = 18	1.40 (−0.12–4.35)	0.60 (−2.30–4.50)	1.80 (0.60–3.90)	0.717
ΔLS-VTQ (1M − 1W), *n* = 20	−0.28 (−0.43–−0.04)	−0.28 (−0.48–0.02)	−0.29 (−0.40–−0.10)	0.820
ΔSS-VTQ (1M − 1W), *n* = 20	−0.00 (−0.32–0.26)	−0.13 (−0.62–−0.04)	0.25 (−0.05–0.40)	0.013

ΔLS-TE was available in 18 patients because measurements could not be obtained in two patients due to ascites. ΔLS-VTQ and ΔSS-VTQ were available in all 20 patients. Continuous variables are expressed as median (interquartile range [IQR]) and were compared using the Mann–Whitney U test. A two-sided *p* value < 0.05 was considered statistically significant. Abbreviations: LS-TE, liver stiffness measured by transient elastography; LS-VTQ, liver stiffness measured by Virtual Touch Quantification; SS-VTQ, spleen stiffness measured by Virtual Touch Quantification.

## Data Availability

The data supporting the findings of this study are not publicly available because of institutional and ethical restrictions but are available from the corresponding author upon reasonable request and with permission from the institution.

## Supplementary Materials

The following supporting information can be downloaded at: https://www.mdpi.com/article/10.3390/jcm15207868/s1. Supplementary Figure S1. Liver stiffness at 1 month after endoscopic treatment in the recurrence and non-recurrence groups. (A) Liver stiffness measured by transient elastography (LS-TE). (B) Liver stiffness measured by Virtual Touch Quantification (LS-VTQ). Open circles indicate the non-recurrence group, and closed circles indicate the recurrence group. Blue and red lines represent the median and interquartile range (IQR) for the non-recurrence and recurrence groups, respectively. Between-group comparisons were performed using the Mann–Whitney U test. Supplementary Figure S2. Individual changes in splenic index from baseline to 1 year after treatment. Individual changes in the splenic index from baseline (Pre) to 1 year (1Y) after treatment are shown. Each line represents an individual patient. Blue and red lines indicate patients without recurrence and with recurrence, respectively. Nineteen patients were included in this analysis; one patient who underwent partial splenic embolization (PSE) was excluded. Supplementary Table S1. Paraesophageal veins and portosystemic shunts according to esophageal variceal recurrence. Categorical variables are expressed as numbers and were compared using Fisher’s exact test. A two-sided *p* value < 0.05 was considered statistically significant.

## Abbreviations

The following abbreviations are used in this manuscript:

EV	esophageal varices;
LS	liver stiffness;
LS-TE	liver stiffness measured by transient elastography;
LS-VTQ	liver stiffness measured by Virtual Touch Quantification;
SS	spleen stiffness;
SS-VTQ	spleen stiffness measured by Virtual Touch Quantification;
TE	transient elastography;
VTQ	Virtual Touch Quantification.
