# Supplementary material for: Early Changes in Spleen Stiffness After Endoscopic Treatment Are Associated with Esophageal Variceal Recurrence: A Pilot Study
Source: J Clin Med. 2026 Oct 12;15(20):7868. doi: 10.3390/jcm15207868 (PMC13705358; doi:10.3390/jcm15207868)
Supplement: Supplementary file 1 [file jcm-15-07868-s001.zip › Table S1.pdf]

Table S1. Paraesophageal veins and portosystemic shunts according to esophageal variceal recurrence

CT finding	**Non-recurrence group (n=9)**	**Recurrence group (n=11)**	**P value**
**Paraesophageal vein, n (%)**	6 (66.7)	3 (27.3)	0.175
**Portosystemic shunt, n (%)**	**6 (66.7)**	**1(9.1)**	**0.017**
Spleno-renal shunt, n	3	0	
Paraumbilical shunt, n	3	1	
Gonadal vein shunt, n	3	0	

Categorical variables are expressed as numbers and were compared using Fisher's exact test. A two-sided p value < 0.05 was considered statistically significant.

Abbreviations: LGV, left gastric vein; SGV, short gastric vein.
